# Epstein-Barr virus infection and chronic lymphocytic leukemia: a possible progression factor?

**DOI:** 10.1186/1750-9378-5-22

**Published:** 2010-11-22

**Authors:** Riccardo Dolcetti, Antonino Carbone

**Affiliations:** 1 Cancer Bio-Immunotherapy Unit, CRO - IRCCS, National Cancer Institute, Via Franco Gallini 2, 33081 Aviano, Italy; 2 Pathology Unit, CRO - IRCCS, National Cancer Institute, Via Franco Gallini 2, 33081 Aviano, Italy

## Abstract

Epstein-Barr virus is pathogenically associated with a well defined group of lymphoid and epithelial tumors in which the virus directly drives transformation of infected cells. Recent evidence however indicates that this virus may infect a subpopulation of tumor cells in patients with chronic lymphocytic leukemia (CLL) and EBV infection has been also associated with Richter transformation in a fraction of cases. We herein review available data suggesting a possible role of EBV as a direct or micro-environmental progression factor in a subset of CLL.

## Introduction

The Epstein-Barr virus (EBV) was first identified 40 years ago in cultured Burkitt's lymphoma cells when no human lymphoid cell had ever been maintained in culture [1]. EBV has a world-wide distribution being able to establish a lifelong infection in more than 90% of individuals. Primary infection is usually asymptomatic and only when it is delayed until adolescence or adulthood a benign lymphoproliferative disease, known as infectious mononucleosis (IM), may occur. The main site of EBV persistence *in vivo *is represented by latently infected B cells showing features of resting memory B lymphocytes [2,3]. Under normal circumstances, EBV is able to establish a persistent infection *in vivo *without affecting the behavior of B lymphocytes. To do so, the virus has evolved an elegant strategy based on the subtle exploitation of virtually all aspects of B cell physiology. The final outcome of the interaction between EBV and the infected host is the establishment of a nonpathogenic latent infection of memory B lymphocytes that allows the virus to persist for the lifetime. Evidence accumulated so far, in particular the presence of EBV genomes and the constant expression of viral proteins, strongly support the involvement of EBV in the pathogenesis of a wide spectrum of human malignancies. These include lymphomas of B, T and NK cell origin such as the immunoblastic lymphoma of immunosuppressed, endemic Burkitt's lymphoma (BL), Hodgkin's Lymphoma (HL), and some T/NK cell lymphoma, but also carcinomas of the nasopharynx and stomach and leiomyosarcomas arising in organ transplant patients and HIV-infected individuals [4]. EBV-induced immortalization/transformation is mediated by the activity of viral proteins that interfere with crucial cellular pathways controlling growth and/or survival. These viral proteins act cooperatively and may induce different biologic effects in different cellular backgrounds [4]. On the basis of the different pattern of latent EBV genes expressed in EBV-associated tumors, three main types of virus latency have been identified. Latency I is the more restricted form of viral gene expression and characterizes BL, which expresses only the EBV nuclear antigen (EBNA)-1 and the EBV RNAs (EBERs). In contrast, latency III involves the unrestricted expression of all the 6 EBNAs together with the latent membrane proteins (LMP)-1 and LMP-2. This type of latency mainly occurs in the setting of severe immune suppression and characterizes post-transplant and HIV-associated lymphoproliferative disorders, and is usually observed in EBV-immortalized lymphoblastoid cell lines *in vitro*. Latency II is an intermediate form of virus latency in which, besides EBNA-1 and EBERs, only LMP-1 and -2 are expressed. This pattern of EBV gene expression is observed in HL, T/NK cell lymphoma, and nasopharyngeal carcinoma (NPC).

EBV can be considered as the prototype of oncogenic viruses that behave as direct transforming agents. In fact, in classical EBV-associated tumors, the virus genome is present in virtually all neoplastic cells, which show the expression of viral RNAs and proteins that variously contribute to the induction of the transformed phenotype. On the basis of these features and of the strict association with distinct tumor types, EBV has been classified as a group I carcinogen. An additional compelling factor is the presence of homogeneous (clonal) EBV episomes detected with the use of the virus termini assay in several EBV-related tumors (HL, NPC, BL) as well as in some pre-neoplastic lesions. These findings suggest that these tumors develop from a single cell that was infected by EBV before the outgrowth and are consistent with a role for EBV in the early phases of tumor development.

Besides the well defined group of tumors pathogenically associated with EBV according to the criteria mentioned above, the presence of this herpesvirus has been variably detected in a broad spectrum of other tumors for which a causal role of EBV seems unlikely. These tumors include also chronic lymphocytic leukemia. We herein briefly review available data suggesting a possible role of EBV as a direct or microenvironmental progression factor in a fraction of CLL.

## Chronic lymphocytic leukemia and Richter's syndrome

Chronic lymphocytic leukemia (CLL) is the most common type of adult leukemia in the United States and Western Europe. CLL cells are small lymphoid B cells with scant cytoplasm having a regular outline. Nuclei contain clumped chromatin and nucleoli are usually absent. On bone marrow and peripheral blood smears the CLL variant with increased prolymphocytes (CLL/PLL), consists of more than 10%, but less than 55% prolymphocytes. Bone marrow histologic pattern may be nodular, interstitial, diffuse, or a combination of the three. These patterns correlate with prognosis [5]. CLL cells express surface IgM or IgM and IgD, CD5, CD19, and CD23. Ig genes are rearranged. 40-50% of cases are un-mutated and 50-60% show somatic hypermutations. There is a group of genes that distinguishes the two genetic subytpes. ZAP-70 is among the genes whose expression is associated with an IgHV un-mutated CLL genotype [5]. About 50% of CLL show 13q deletion, about 20% trisomy 12 and, less commonly, other genomic abnormalities. Low stage patients with mutated CLL have a better prognosis than those with un-mutated CLL. ZAP-70 and CD38 expression of are associated with a poor prognosis [5].

**Figure 1:**
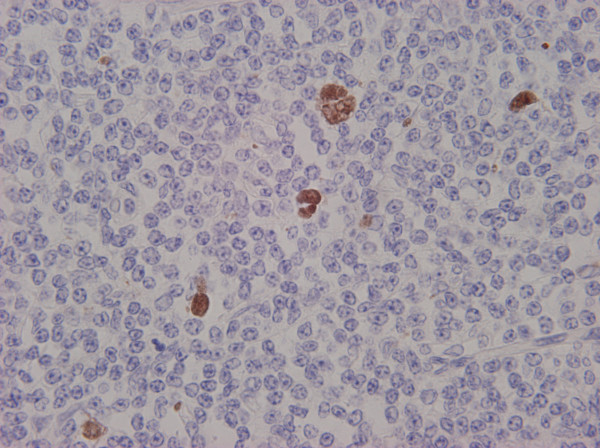
**Richter's transformation of a CLL case**. *In situ *EBER hybridization shows nuclear staining in giant Hodgkin/Reed-Sternberg-like cells (660×).

A small fraction (approximately 2-8%) of patients with CLL develop diffuse large B-cell lymphoma (DLBCL). This event has been termed Richter's syndrome or Richter's transformation [6] and is associated with poor response to treatment and low survival times [5]. Richter's syndrome is histologically characterized by confluent sheets of large cells that may resemble centroblast- or immunoblast-like cells. The majority of the reported DLBCL occurring in patients with CLL are clonally related to the previous CLL [7,8]. An interesting finding is the occurrence of scattered Hodgkin and Reed Sternberg (HRS)-like cells in the background. In contrast to true Hodgkin lymphoma arising in CLL patients, the reactive typical background is absent and the HRS-like cells are surrounded by neoplastic B-cells. These HRS-like cells show evidence of EBV infection (Figure 1).

## EBV and chronic lymphocytic leukemia

EBV infection is only infrequently detected in CLL by conventional diagnostic approaches. This is consistent with *in vitro *findings indicating that CLL cells do not regularly become activated or immortalized after exposure to EBV [9], although this can be achieved following cytokine activation [10]. It has been shown, in particular, that the combined treatment with *Staphylococcus aureus *Cowan I (SAC), MP6-thioredoxin, and interleukin 2 can render CLL cells susceptible to EBV infection *in vitro *[10]. Therefore, although basally refractory, CLL cells may become occasionally infected by EBV *in vivo *provided that mitogenic and activating signals are available within the microenvironment. In CLL cells, however, the expression of the EBV-encoded nuclear proteins EBNAs is not followed by entrance into the cell cycle. In fact, unlike what observed in normal B lymphocytes, EBV-infected CLL cells remain resting and do not express c-myc, cyclin D2, or pRb, whereas the p27 cell cycle inhibitor is not down-regulated [11].

In CLL cases showing EBV infection, EBV markers are however detectable only in a subpopulation of tumor cells [12,13]. Notably, several studies have reported that expression of EBERs detected by *in situ *hybridization correlated with progressive or accelerated clinical courses [13-16]. EBERs are small non-coding RNAs abundantly expressed in latently infected cells that play critical role in B cell transformation and induction of resistance to apoptosis [17], and may therefore directly contribute to CLL progression. Further characterization of EBV infection in this setting allowed the demonstration of LMP-1 mRNA expression in isolated CLL cells but not in normal resting B lymphocytes [18]. More recently, LMP-1 transcriptional activity was detected in a significant proportion of CLL cases (14% *vs*. 1% of healthy controls) [19]. Notably, CLL patients showing LMP-1 mRNA expression had a higher extent of marrow involvement [19]. Although a confirmation that LMP-1 is expressed also at the protein level is still lacking, available evidence is consistent with a possible role for EBV in the malignant progression of the disease. EBV infection has been also associated with a fraction of CLL transformation to diffuse large cell lymphoma or Richter syndrome, particularly in cases displaying a high number of Reed-Sternberg-like cells [15,20-23]. Cases of CLL transformation to HL have been also reported [23,24]. Considering that LMP-1 drives the morphologic and transcriptional changes characteristic of Hodgkin/Reed-Sternberg cells [25], the detection of LMP-1 expression in tumor cells of a fraction of CLL cases is particularly intriguing and deserves further investigation. In some, but not all cases, the malignant transformation involves cells originating from the CLL clone, as shown by immunoglobulin gene rearrangement studies [26-29], further supporting the role of secondary EBV infection as a possible cyto-morphologic and clinical progression factor for CLL. In clonally distinct cases, the immune suppression induced by drugs such as fludarabine or anti-CD52 antibodies may favor the outgrowth of an unrelated, EBV-infected B cell clone that may lead to a clinically aggressive disease resembling EBV-associated lymphoproliferations of immunosuppressed patients. These cases should be distinguished from Richter syndrome, probably representing a novel type of immunodeficiency-related lymphoma.

Besides behaving as a direct drive of neoplastic progression in EBV-infected CLL cells, the presence of EBV within tumor microenvironment could also indirectly contribute to the malignant evolution of the disease. Indeed, EBV may infect a subpopulation of CLL cells and/or may be carried by bystander normal B lymphocytes. EBV infection and, particularly, LMP-1 expression, by CLL cells may promote angiogenesis by inducing IL-8 [30], and increasing the production of vascular endothelial growth factor [31]. Moreover, EBV may induce/enhance the production of cytokines, such as cellular IL-10 (cIL-10), able to promote B cell proliferation and inhibit T cell responses [32]. Notably, serum cIL-10 levels are increased in CLL patients and correlate with adverse disease features and short survival [33]. Intriguingly, assays measuring both EBV-derived (viral IL-10) and human cIL-10 yielded higher values than assays specifically quantifying human cIL-10 [33]. These findings indirectly indicate that, in a fraction of CLL cases, EBV infection is not silent, being associated with enhanced production of viral IL-10. EBV may also contribute to a local immune suppression through the production of hydrophobic peptides derived from the first transmembrane domain of LMP-1 [34]. These peptides, in fact, potently inhibit both cytotoxic T lymphocyte and natural killer cell responses *in vitro *[34].

## Concluding remarks and future perspectives

In CLL, evidence for EBV infection was obtained only in a subset of cases and, notably, only in a variable fraction of neoplastic cells. These findings contrast with the constant presence of EBV genome in all tumor cells of malignancies pathogenically linked to the virus and argue against a causal role for EBV in the early phases of CLL development. Although a partial loss of viral genomes can not be formally excluded in these cases, this possibility appears however unlikely. Moreover, the fact that viral products necessary to sustain the transformed phenotype may be expressed only by a fraction of tumor cells further weakens the hypothesis of a pathogenic involvement of EBV in this tumor. Although further studies are required to elucidate this complex and still debated issue, available evidence briefly reviewed herein is consistent with a possible role of EBV infection as a secondary event occurring after cell transformation and affecting a subpopulation of CLL cells in which the virus may promote the evolution to a more malignant phenotype. The possible prognostic role of EBV-driven production of factors able to promote CLL cell growth, angiogenesis and to inhibit tumor-specific immune responses deserves to be carefully investigated by prospective studies. If such progression mechanisms will be conclusively demonstrated, the use of antagonists or inhibitors of molecules produced by EBV-infected cells could represent a potentially attractive therapeutic approach.

## Competing interests

The authors declare that they have no competing interests.

## Authors' contributions

RD and AC conceived and wrote the review manuscript. All authors read and approved the final manuscript.
